# Is Conception by Means In Vitro Fertilization Associated With Increased Risk of Antenatal Anxiety and Depression?

**DOI:** 10.7759/cureus.36659

**Published:** 2023-03-25

**Authors:** Olga Arvanitidou, Dimitrios Rafail Kalaitzopoulos, Nicolas Samartzis, Apostolos Athanasiadis, Ioanna Ierodiakonou-Benou, Angelos Daniilidis

**Affiliations:** 1 Third Department of Obstetrics and Gynaecology, Aristotle University of Thessaloniki, Thessaloniki, GRC; 2 Gynecology, Zurich University Hospital, Zurich, CHE; 3 Department of Gynecology and Obstetrics, Cantonal Hospital Schaffhausen, Schaffhausen, CHE; 4 Second Department of Obstetrics and Gynaecology, Aristotle University of Thessaloniki, Thessaloniki, GRC; 5 Third University Department of Psychiatry, Aristotle University of Thessaloniki, Thessaloniki, GRC; 6 Second Department of Obstetrics and Gynecology, School of Medicine, Hippokration General Hospital, Aristotle University of Thessaloniki, Thessaloniki, GRC

**Keywords:** mental health, infertility, anxiety, depression, ivf

## Abstract

Objectives

Mental health during pregnancy is a very important public health issue with negative effects on both maternal and child outcomes. The aim of our study is to examine the possible association between conception via in vitro fertilization (IVF) and anxiety or depression during the third pregnancy trimester in the Greek population during the years of financial crisis.

Materials and Methods

This single-center prospective cohort study was conducted in a tertiary university hospital during the period 2017-2018. Pregnant women attending the Antenatal Care Program between 30th-32nd gestational week were asked to complete Hamilton Anxiety Rating Scale (HAM-A) and Beck Depression Inventory (BDI). A propensity score match for 10 variables was conducted in a 1:3 ratio.

Results

Of the 521 eligible patients, 446 women were included in our study. Four hundred fourteen of them conceived spontaneously, and 32 via IVF. After propensity score matching, 76 remained in the analysis, of whom 57 conceived spontaneously and 19 with IVF. The IVF group had a higher rate of anxiety (18.8%) and a lower rate of depression (9.4%) than the spontaneous conception group (13.5% and 13.5%, respectively), but the differences were not statistically significant before and after propensity score matching.

Conclusion

Our study showed that pregnancies after IVF had a higher incidence of antenatal anxiety and a lower incidence of antenatal depression in comparison to pregnancies that were conceived naturally, although the differences did not reach statistical significance.

## Introduction

Mental health during pregnancy is a very important public health issue with negative effects on both maternal and child outcomes [1]. Two of the most common mental disorders during pregnancy; and postpartum are anxiety and depression. The incidence of maternal anxiety symptoms varies according to the time of the diagnosis, with a frequency of 18.2% (95% CI 13.6-22.8) in the first, 19.1% (95% CI 15.9-22.4) in the second, and 24.6% (95% CI 21.2-28.0) in the third trimester. The overall prevalence of any anxiety disorder is 15.2% (95% CI 9.0-21.4) and 4.1% (95% CI 1.9-6.2) for a generalized anxiety disorder [2]. There has been reported an association between maternal anxiety and diverse adverse outcomes such as preterm birth, lower Apgar scores, cognitive disorders in childhood, and increased risk of maternal suicide [2].

According to an umbrella review, the prevalence of antenatal depression ranged from 15% to 65%, with prominent risk factors being exposed to different forms of abuse and violence, a lack of support, and a personal or family history of mental disorder [3]. The diagnosis of antenatal depression during pregnancy is associated with an increased risk of maternal complications such as preeclampsia and postpartum depression, premature rupture of membranes, and hemorrhage and adverse outcomes on the neurological, behavioral, and emotional development of the children [3]. Because of its effectiveness and safety, in vitro fertilization (IVF) is the most used method of assisted reproductive treatment (ART). It has been previously reported that IVF could be associated with psychological and emotional stress because of the high costs, complicated procedures with daily injections and blood samples, oocyte retrieval, and high failure rates [4]. Our study aims to examine the possible association between conception via IVF and anxiety or depression during the third pregnancy trimester in the Greek population during the years of the financial crisis.

## Materials and methods

Study design

A single-center prospective, observational cohort study was conducted in a tertiary university hospital during the period 2017-2018. The study protocol was approved by the Institutional Review Board and Ethical Committee. Informed consent was obtained from all participants during the process.

Inclusion and exclusion criteria

Pregnant women attending the Antenatal Care Program (ACP) were found eligible to participate in this study. ACP is run by midwives with the primary goal of educating and supporting women and couples about pregnancy, labor, the postnatal period, and breastfeeding. Most antenatal classes start around 8-10 weeks before the estimated date of labor, frequently between the 30th-32nd gestational week. Classes are normally held once a week for around 2-3 hours. Pregnant women were excluded from our study if they met one of the following exclusion criteria: 1) maternal age <18 years or >50 years; 2) cardiovascular pathology at the time of study inclusion; 3) history of mental and psychiatric disorders; 4) history of alcohol or drug abuse; 5) history of diagnosed congenital anomalies in the previous pregnancies.

Data collection

Following an interview, eligible participants provided sociodemographic information (type of residence, religion, medical insurance, level of education, income, employment status), previous medical history, and information about their current pregnancy. The women who fulfilled the inclusion criteria were asked to complete Greek versions of both the Hamilton Anxiety Rating Scale (HAM-A) and the Beck Depression Inventory (BDI). The HAM-A is a 14-item self-report questionnaire with ratings ranging from 0 to 4 based on the severity of the symptoms. According to HAM-A, a score ranging between 0-17 indicates mild anxiety, 18-24 mild to moderate anxiety, 25-30 moderate to severe, and 30 and above severe anxiety. The BDI is a 21-item self-report questionnaire; every item is rated from 0 to 3 according to the severity of the symptoms, and the total score of 17-20 indicates borderline clinical depression, 21-30 moderate depression, 31-40 severe depression, and over 40 extreme depression. The Greek version of the BDI questionnaire was validated by Fountoulakis et al., while the Greek version of the HAM-A has, to the best of our knowledge, not been validated [5].

The questionnaire was completed in the presence of the main investigator in order to provide explanations to participants. Information about delivery and postpartum outcomes, such as delivery modus, place of delivery, neonatal weight, neonatal outcome, and breastfeeding, were collected after reviewing the medical records.

Statistical analysis

Sample Size Estimation

To the best of our knowledge, there is no published study on the Greek population with a similar design. According to a previous study in infertile women during pregnancy, anxiety was diagnosed in 66.6%, while depression was diagnosed in 31.2% [6]. According to the sample size calculation with a power of 80% and a level of confidence of 95%, the sample size should be at least 110 women per group for anxiety and 23 women per group at least for depression. According to older data from our clinic, about 6% of the live births are after IVF/ICSI; thus, we need to enroll a minimum of 384 patients to detect the difference in antenatal depression between the two groups.

Data Analysis

All the participants were divided into two groups, i.e., those who conceived after IVF/ICSI and those who conceived spontaneously. To compare baseline and clinical characteristics, the Pearson chi-square test for categorical variables and the Mann-Whitney U test for continuous variables were used. 

A propensity score matching for 10 variables was conducted in a 1:3 ratio after using the nearest-neighbor matching algorithm with caliper widths equal to 0.2. The potential role of baseline characteristics as confounding factors was examined with multivariate logistic regression analysis. Data analysis was conducted using IBM Corp. Released 2020. IBM SPSS Statistics for Windows, Version 27.0. Armonk, NY: IBM Corp. A p-value of <0.05 was set as the level of statistical significance.

## Results

Patients population and baseline characteristics

**Figure 1:**
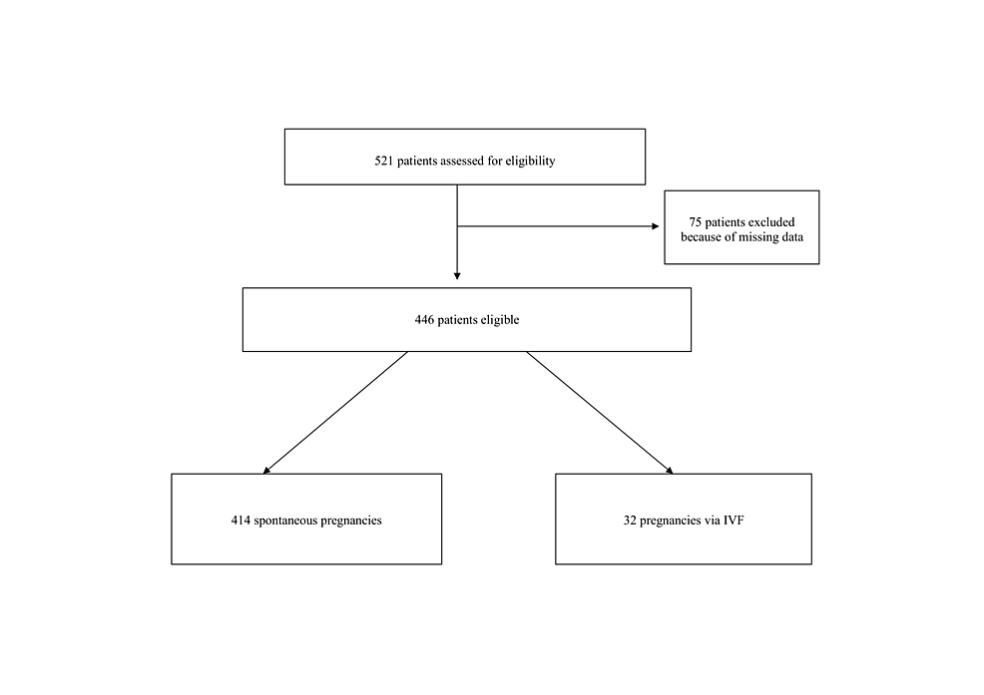
Flowchart

Of the 521 eligible patients, 446 women were included in our study (Figure 1). Four hundred and fourteen of them conceived spontaneously, and 32 after IVF. Between the two groups, there were significant differences in age, religion, ethnicity, insurance type, pregnancy type, and follow-up outcomes in delivery, neonatal weight, and breastfeeding. After propensity score matching, 76 remained in the analysis, of whom 57 conceived spontaneously and 19 with IVF, without any significant differences in baseline characteristics (Table 1).

**Table 1:** Baseline characteristics and pregnancy outcomes before and after propensity score matching *Mann-Whitney-U, chi-square for all other outcomes

		Before propensity matching			After propensity matching		
		IVF	Spontaneous		IVF	Spontaneous	
		n=32	n=414	p-value	n=19	n=57	p-value
Age	<20	0% (n=0)	1.0% (n=4)	<0.001	0% (n=0)	0% (n=0)	0.989
	20-25	0% (n=0)	6.3% (n=26)		0% (n=0)	0% (n=0)	
	26-30	3.1% (n=1)	29.5% (n=122)		5.3% (n=1)	7.0% (n=4)	
	31-35	34.4% (n=11)	43.5% (n=180)		31.6% (n=6)	29.8% (n=17)	
	36-40	43.8% (n=14)	16.4% (n=68)		47.4% (n=9)	49.1% (n=28)	
	>40	18.8% (n=6)	3.4% (n=14)		15.8% (n=3)	14.0% (n=8)	
Residency	rural	0% (n=0)	4.6% (n=19)	0.45	0% (n=0)	7.0% (n=4)	0.49
	small city	12.5% (n=4)	10.6% (n=44)		10.5% (n=2)	8.8% (n=5)	
	city	87.5% (n=28)	84.8% (n=351)		89.5% (n=17)	84.2% (n=48)	
Education	Secondary school	3.1% (n=1)	1.2% (n=5)	0.60	5.3% (n=1)	0% (n=0)	0.324
	High-school	15.6% (n=5)	22.0% (n=91)		26.3% (n=5)	21.1% (n=12)	
	university	40.6% (n=13)	43.2% (n=179)		31.6 (n=6)	40.4% (n=23)	
	MSc/PhD	40.6% (n=13)	33.6% (n=139)		36.8% (n=7)	38.6 (n=22)	
Income per year (euro)	<5000	3.1% (n=1)	6.0% (n=25)	0.26	0% (n=0)	3.5% (n=2)	0.753
	5000-10000	6.3% (n=2)	18.6% (n=77)		10.5% (n=2)	22.8% (n=13)	
	10000-150000	31.3% (n=10)	27.3% (n=113)		31.6% (n=6)	21.1% (n=12)	
	15000-20000	21.9% (n=7)	24.2% (n=100)		21.1% (n=4)	21.1% (n=12)	
	20000-25000	18.8% (n=6)	15.0% (n=62)		15.8% (n=3)	15.8% (n=9)	
	>25000	18.8% (n=6)	8.9% (n=37)		21.1% (n=4)	15.8% (n=9)	
Religion	Christianism	87.5% (n=28)	94.2% (n=390)	0.03	89.5% (n=17)	94.7% (n=54)	0.426
	Islam	3.1% (n=1)	0.2% (n=1)		0% (n=0)	0% (n=0)	
	other	3.1% (n=1)	5.1% (n=21)		0% (n=0)	1.8% (n=1)	
	without	6.2% (n=2)	0.5% (n=2)		10.5% (n=2)	3.5% (n=2)	
Ethnicity	Europe	96.9% (n=31)	99.5% (n=412)	0.001	100% (n=19)	100% (n=57)	n/a
	Africa	3.1% (n=1)	0% (n=0)		0% (n=0)	0% (n=0)	
	Asia	0% (n=0)	0.5% (n=2)		0% (n=0)	0% (n=0)	
Insurance	Public	87.5% (n=28)	87.9% (n=364)	0.002	94.7% (n=18)	96.5% (n=55)	0.734
	Private	0% (n=0)	4.8% (n=20)		0% (n=0)	0% (n=0)	
	Other	9.4% (n=3)	7.2% (n=30)		5.3% (n=1)	3.5% (n=2)	
	No Insurance	3.1% (n=1)	0% (n=0)		0% (n=0)	0% (n=0)	
Profession	Private sector	37.5% (n=12)	46.4% (n=192)	0.06	31.6% (n=6)	49.1% (n=28)	0.191
	Public sector	34.4% (n=11)	13.3% (n=55)		36.8% (n=7)	14.0% (n=8)	
	Self-employed	6.3% (n=2)	10.9% (n=45)		10.5% (n=2)	8.8% (n=5)	
	Student	0% (n=0)	0.2% (n=1)		0% (n=0)	0% (n=0)	
	Academic	6.3% (n=2)	9.9% (n=41)		5.3% (n=1)	15.8% (n=9)	
	Unemployed	15.6% (n=5)	19.3% (n=80)		15.8% (n=3)	12.3% (n=7)	
Parity	1	68.8% (n=22)	75.1% (n=311)	0.58	73.7% (n=14)	54.4% (n=31)	0.321
	2	28.1% (n=9)	20.5% (n=85)		21.1% (n=4)	33.3% (n=19)	
	3	3.1% (n=1)	4.3% (n=18)		5.3% (n=1)	12.3% (n=7)	
Pregnancy	Single	75% (n=24)	99% (n=410)	<0.001	100% (n=19)	98.2% (n=56)	0.561
	Twin	25% (n=8)	1% (n=4)		0% (n=0)	1.8% (n=1)	
Delivery	Spontaneous	15.6 % (n=5)	58.4% (n=242)	<0.001	21.1% (n=4)	24.6% (n=14)	0.046
	Operative Vaginal	6.3% (n=2)	7.2% (n=30)		10.5% (n=2)	0% (n=0)	
	C-Section	78.1% (n=25)	34.3% (n=142)		68.4% (n=13)	75.4% (n=43)	
Preterm birth		15.6% (n=5)	9.9% (n=41)	<0.001	10.5% (n=2)	28.1% (n=16)	0.119
Perinatal death		0% (n=0)	0.5% (n=2)	1	0% (n=0)	1.8% (n=1)	0.561
Neonatal weight		2853 (504)	3199 (467)	<0.001*	3029 (445)	2936 (620)	0.905*
Rooming		65.6% (n=21)	56.0% (n=232)	0.36	36.8% (n=7)	26.3% (n=15)	0.381
Breast feeding		46.9% (n=15)	68.8% (n=285)	0.017	47.4% (n=9)	45.6% (n=26)	0.894
Place of delivery	Public Hospital	21.9% (n=7)	33.6% (n=139)	0.36	15.8% (n=3)	31.6% (n=18)	0.183
	Private Hospital	78.1% (n=25)	65.9% (n=273)		84.2% (n=16)	68.4% (n=39)	
	Home	0% (n=0)	0.5% (n=2)		0% (n=0)	0% (n=0)	

Primary outcomes

Women who had IVF pregnancies had higher rates of anxiety (18.8%) and lower rates of depression (9.4%) than women who conceived spontaneously (13.5% and 13.5%, respectively), though the differences were not statistically significant (p=0.411 and p=0.504). There were also no statistically significant differences between the HAM-A and BDI total scores of the two groups compared as far as HAM-A and BDI are concerned. After matching the two groups with propensity scores for 10 variables, the tendency of higher anxiety and lower depression in the IVF group remained, but the differences were still non-significant. The IVF group had a significantly higher HAM-A score after the propensity score, but the difference in BDI total score remained non-significant. Women with both depression and anxiety were comparable in both groups before and after matching (Table 2).

**Table 2:** Primary outcomes before and after propensity score matching *Mann-Whitney-U, chi-square for all other outcomes

	Before propensity matching			After propensity matching		
	IVF	Spontaneous		IVF	Spontaneous	
	n=32	n=414	p-value	n=19	n=57	p-value
Anxiety	18.8% (n=6)	13.5 (n=56)	0.411	15.8% (n=3)	7.0% (n=4)	0.252
HAM-A	12.41 (6.53)	10.78 (6.82)	0.103	11.95 (5.20)	9.23 (5.80)	0.017
Depression	9.4% (n=3)	13.5% (n=56)	0.504	5.3% (n=1)	22.8% (n=13)	0.08
BDI	8.78 (6.12)	9.90 (6.36)	0.272	8.47 (4.26)	11.21 (6.54)	0.139
Anxiety and Depression	6.3% (n=2)	5.8% (n=24)	0.916	0% (n=0)	3.5% (n=2)	0.408

The role of six cofounders on total BDI and HAM-A scores was examined with simple linear regression (Table 3). Age was the only confounder that had a significant association with BDI score both before and after matching the two groups (p=0.025 and p=0.016, respectively), indicating that younger women are more likely to have a higher BDI score. As far as the HAM-A score is concerned, the only significant confounder before propensity score matching was income, with a reverse association with the HAM-A score. This association was not significant after propensity score matching. The only cofounder which showed a significant association after propensity score matching was the pregnancy type, showing that twin pregnancies were associated with a higher HAM-A score.

**Table 3:** Logistic regression antenatal depression (a) and antenatal anxiety (b).

	unmatched		matched with propensity score	
a)	linear regression OR (95% CI)	p-value	linear regression OR (95% CI)	p-value
Age	-2.25 (-1.48, -0.10)	0.025	-2.48 (-4.05, -0.44)	0.016
Residency	0.72 (-0.78, 1.69)	0.472	0.63 (-1.82, 3.50)	0.528
Education	-1.49 (-1.50, 0.20)	0.137	1.16 (-0.83, 3.12)	0.251
Income in euro per year	0.45 (-0.37, 0.59)	0.653	-0.20 (-1.24, 1.02)	0.842
Parity	3.06 (0.64, 2.94)	0.002	2.11 (0.12, 4.33)	0.039
Pregnancy type	1.09 (-1.78, 6.15)	0.278	1.67 (-1.94, 22.07)	0.099

	unmatched		matched with propensity score	
b)	linear regression OR (95% CI)	p-value	linear regression OR (95% CI)	p-value
Age	-0.99 (-1.11, 0.37)	0.321	-0.67 (-2.30,1.14)	0.506
Residency	0.29 (-1.13, 1.52)	0.771	0.27 (-2.19, 2.87)	0.790
Education	0.17 (-0.84, 1.00)	0.864	0.99 (-0.95, 2.80)	0.327
Income in euro per year	-2.54 (-1.19, -0.15)	0.011	-1.56 (-1.90, 0.24)	0.124
Parity	0.42 (-0.97, 1.50)	0.671	0.80 (-1.20, 2.80)	0.425
Pregnancy type	1.50 (-1.01, 7.49)	0.135	2.99 (5.61, 28.39)	0.004

## Discussion

The results of our prospective cohort study showed that women who conceived after IVF have an increased risk of antenatal anxiety and decreased risk of antenatal depression between the 30th and 32nd gestational week in comparison with women who conceived spontaneously, although both associations were not significant before and after propensity score matching to account for potential confounders [7]. After propensity score matching, the IVF group had a significantly higher total HAM-A score.

Despite the well-known effect of infertility and its treatment on mental health, only a few studies have examined the association between the method of conception and anxiety or depression during pregnancy [8]. A recent review of the available literature on the subject concluded that the evidence does not support IVF as a factor associated with perinatal affective symptoms [8]. In terms of perinatal depression, a large longitudinal study from Sweden with 3283 women found no link between IVF and natural conception [9], while smaller studies found that IVF groups have both higher [10] and lower rates of antenatal depression [11]. The high heterogeneity of the above studies could be attributed to the different time points and methods (questionnaires, interviews) of the assessment.

A study that examined depression during pregnancy at different time points in both women who conceived via ART and naturally showed that the prevalence was similar, although the ART group had different rates of change in depressive symptoms through gestation [8]. Another meta-analysis of eight studies found a comparable prevalence of postpartum depression in the IVF and natural conception groups [12]. 

According to an umbrella review [3], the most commonly reported risk factors for antenatal depression are psychological factors such as a history of abuse, a lack of social support network, and a history of personal or family mental disorder, pregnancy-related factors such as an unplanned or unwanted pregnancy, and a history or current pregnancy with adverse outcomes such as hyperemesis, preterm birth, stillbirth, infant death after delivery, and cesarean section delivery, and socioeconomic factors. In our study, linear regression analysis showed a significant association between antenatal depression and age before and after propensity score matching. The remaining characteristics (residency, education level, yearly income, parity, and the number of fetuses) were not significantly associated with antenatal depression. A previous study found that IVF was associated with higher anxiety rates, particularly among women who had previously miscarried [13]. Women are more emotionally affected by anxiety during the first months of infertility treatment [14]. Longer infertility duration or a history of treatment failure had a higher risk of developing antenatal anxiety [15]. Women who became pregnancy after IVF may be overly concerned about the possibility of losing their pregnancy [16], and the possible role of diverse stress biomarkers with different concentrations between pregnant after IVF and spontaneous conception is also discussed [17]. Data for women conceiving with IVF showed that this population tends to have less anxiety with the progression of the pregnancy [18], possibly because of the resistance to stress developed after the IVF procedure [19]. A meta-analysis, on the other hand, found that in the general population, the prevalence of self-reported anxiety during pregnancy is higher in the third pregnancy trimester (24.6%) [2]. The prevalence of mental health issues antenatal and postpartum seems to differ between countries with differences in financial status [11]. A previous study in a Greek public hospital before the COVID-19 pandemic, which examined 163 pregnant women between 11 and 26 gestational weeks, using the Center for Epidemiologic Studies-Depression scale (CES-D) and the State-Trait Anxiety Inventory (STAI-X), found that women who conceived after IVF had a significantly higher level of anxiety, while low annual income was associated with antenatal depression [13]. A recent study during the COVID-19 pandemic showed a similar prevalence of antenatal depression, 13.5%, in comparison to our population [20]. However, anxiety seems to be higher during the pandemic, with a prevalence of 34.1%, according to the above study, and 24.8%, according to another study [21]. The flowchart of the study is shown in Figure 1.

Strengths and limitations

The prospective design and the large sample size are two of the main strengths of our study. The presence of the main investigator during the completion of interviews and questionnaires to give some explanations needed resulted in the inclusion of 86% of the eligible patients. In addition, the propensity score matching helped to balance the differences between the heterogeneous groups. On the other hand, there are some limitations to our study. A single-center study could have some selection bias, although our unit covers a large population in northern Greece with a representative number of low-risk pregnancies. The lack of some relevant information about the history of infertility, such as the history of miscarriage, duration of infertility, and other potential confounders, is also a certain limitation [22]. Last but not least, the self-reported type of our study could also hide a recall bias.

## Conclusions

Our study showed that pregnancies after IVF had a higher incidence of antenatal anxiety and a lower incidence of antenatal depression compared to naturally conceived pregnancies, although the differences were not statistically significant. More studies are needed to extract safe conclusions about the association between mental health disorders and conception via IVF.
